# Metabolic Dysfunctions in Amyotrophic Lateral Sclerosis Pathogenesis and Potential Metabolic Treatments

**DOI:** 10.3389/fnins.2016.00611

**Published:** 2017-01-10

**Authors:** Tesfaye W. Tefera, Karin Borges

**Affiliations:** Laboratory for Neurological Disorders and Metabolism, School of Biomedical Sciences, Department of Pharmacology, The University of QueenslandBrisbane, QLD, Australia

**Keywords:** amyotrophic lateral sclerosis, energy metabolism, medium chain fatty acids, mitochondria, metabolic treatment

## Abstract

Amyotrophic lateral sclerosis (ALS) is a fatal neurodegenerative disease primarily characterized by loss of motor neurons in brain and spinal cord. The death of motor neurons leads to denervation of muscle which in turn causes muscle weakness and paralysis, decreased respiratory function and eventually death. Growing evidence indicates disturbances in energy metabolism in patients with ALS and animal models of ALS, which are likely to contribute to disease progression. Particularly, defects in glucose metabolism and mitochondrial dysfunction limit the availability of ATP to CNS tissues and muscle. Several metabolic approaches improving mitochondrial function have been investigated *in vitro* and *in vivo* and showed varying effects in ALS. The effects of metabolic approaches in ALS models encompass delays in onset of motor symptoms, protection of motor neurons and extension of survival, which signifies an important role of metabolism in the pathogenesis of the disease. There is now an urgent need to test metabolic approaches in controlled clinical trials. In addition, more detailed studies to better characterize the abnormalities in energy metabolism in patients with ALS and ALS models are necessary to develop metabolically targeted effective therapies that can slow the progression of the disease and prolong life for patients with ALS.

## Introduction

Amyotrophic lateral sclerosis (ALS) is a complex multi-pathogenic neurodegenerative disorder primarily characterized by the loss of upper motor neurons in the brain and lower motor neurons in the spinal cord and the brain stem (Robberecht and Philips, 2013). As a result of this neuronal loss, motor function declines progressively which then leads to impairments in respiratory function and eventually death within 2–5 years of diagnosis (Kiernan et al., 2011). The exact mechanisms underlying the selective loss of motor neurons in the disease are not yet known. However, several mechanisms appear to contribute, including glutamate excitotoxicity (Rothstein et al., 1990), oxidative stress (Barber et al., 2006), protein aggregation (Rosen et al., 1993; Bruijn et al., 1998), cytoskeletal and axonal transport abnormalities (Julien and Beaulieu, 2000), inflammation (Julien and Beaulieu, 2000), hyperexcitability (Zanette et al., 2002; Vucic and Kiernan, 2006; Vucic et al., 2008) and altered energy metabolism (Dupuis et al., 2004, 2011). It is believed that several of these mechanisms are interconnected and together are responsible for the progression of the disease. To date, there is only one approved medication available to treat ALS, namely riluzole, which only increases survival by 3 months (Bensimon et al., 1994). Therefore, it is imperative to look for other potential treatments that can halt the progression of the disease and extend life. In this review, we discuss impairments in energy metabolism in ALS and review the effects of metabolic therapies investigated so far in animal models of ALS and/or patients with ALS.

## Energy metabolism disturbances in ALS

There are many lines of evidence for the role of disturbed energy metabolism in ALS pathogenesis. Energy homeostasis, the balance between energy intake and expenditure, appears to be compromised in patients with ALS (Dupuis et al., 2011). Besides, some patients exhibit hypermetabolism (an increased metabolism at rest) (Kasarskis et al., 1996; Desport et al., 2001; Bouteloup et al., 2009; Marin et al., 2011), body weight loss (Körner et al., 2013b) and abnormal metabolism of lipids (Dupuis et al., 2011), which are likely to contribute to disease. These and many studies discussed below reveal problems in energy metabolism in whole tissues and even the whole organism. It is not entirely clear to which extent the metabolic changes found in ALS patients and ALS models only arise as a result of neurodegeneration. Moreover, little is known about the specific metabolic changes within the motor neurons themselves and if they contribute to the degeneration. Other early reviews discuss reasons for the specific degeneration of motor neurons in ALS (Shaw and Eggett, 2000; Cleveland and Rothstein, 2001).

### Specific dysfunctions in energy producing pathways in ALS

Under normal physiological conditions, glucose is the main energy substrate for neuronal tissue and a major fuel for muscles. However, there are indications of defects in the glucose metabolizing pathways in ALS namely in the glycolysis and the TCA cycle pathways in CNS and muscle (Palamiuc et al., 2015; Tefera et al., 2016). In addition, there is decreased glucose uptake in numerous brain regions and spinal cord of animal models and patients with ALS (Dalakas et al., 1987; Hatazawa et al., 1988; Ludolph et al., 1992; Browne et al., 2006; Miyazaki et al., 2012; Cistaro et al., 2014). In muscle, namely the tibialis anterior muscle of the superoxide dismutase 1 (SOD1^G86R^) mice, the activities of the enzyme phosphofructokinase 1 (PFK1), the rate limiting enzyme in glycolysis, were reduced (Palamiuc et al., 2015). Furthermore, in the gastrocnemius muscle of SOD1^G93A^ mice, the activities of the TCA cycle enzyme 2-oxoglutarate dehydrogenase (OGDH) were found to be reduced in the late symptomatic phases of the disease, indicating impairments in the TCA cycle pathway (Tefera et al., 2016). Furthermore, changes in the expression of several genes involved in energy metabolism have been observed in muscle, brain and spinal cord tissues and cells isolated from them. Particularly, genes involved in the mitochondrial electron transport chain are significantly altered (Ferraiuolo et al., 2007, 2011; Lederer et al., 2007; Raman et al., 2015). Mitochondria are the main site of energy production and they are also the main sites for the generation of reactive oxygen species. Compromised mitochondrial function has been recognized for a long time in many neurodegenerative disorders, which is expected to lead to reduced production of ATP and increased oxidative stress (Cozzolino and Carrì, 2012). Similarly in ALS, numerous studies have shown functional and structural abnormalities in mitochondria that give rise to reduced oxidative phosphorylation and subsequent decreased generation of ATP (Jung et al., 2002; Mattiazzi et al., 2002; Menzies et al., 2002; Wiedemann et al., 2002; Browne et al., 2006). In addition, *in vitro* studies showed that mitochondrial dysfunction in astrocytes may lead to generation of free radicals and release of toxic factors that could contribute to motor neuron dysfunction (Nagai et al., 2007; Cassina et al., 2008). In conclusion, pathological changes in the energy metabolizing pathways that lead to deficiency in ATP production and/or utilization may contribute to progression of the disease.

### Role of glial cells in metabolism and ALS

Although ALS is mainly characterized by the selective loss of motor neurons, neighboring non-neuronal cells such as astrocytes (Clement et al., 2003; Nagai et al., 2007), microglia (Beers et al., 2006; Boillée et al., 2006b) and oligodendrocytes (Yamanaka et al., 2008; Lee et al., 2012; Kang et al., 2013) can contribute to the progression of the disease (reviewed by Boillée et al., 2006a). The metabolic coupling between neurons and astrocytes has long been known. During neurotransmission, neurons release glutamate into the synapse which can be taken up by astrocytes. It is then metabolized to glutamine, which can be supplied to neurons to resynthesize glutamate. Many immunohistochemical studies have shown the loss of glutamate transporters in astrocytes in spinal cord and brain of patients with ALS (Rothstein et al., 1995; Fray et al., 1998; Sasaki et al., 2000). Deficiencies in glutamate uptake and the glutamate-glutamine cycle can lead to problems in neuronal signaling and promotes excitotoxicity (reviewed by Danbolt, 2001; Van Den Bosch et al., 2006). In addition, according to the astrocyte-neuron lactate shuttle hypothesis, glutamate stimulated metabolism of glucose in astrocytes produces lactate which can be transferred to neurons to be utilized as fuel (Pellerin and Magistretti, 1994). Altered gene expression of the astrocytic lactate efflux transporter (monocarboxylate transporter 4, SLC16A4) together with reduced lactate levels found in the spinal cord of hSOD1^G93A^ mice further suggest that metabolic interaction between neurons and astrocytes may be disrupted in ALS (Ferraiuolo et al., 2011).

The potential of metabolic coupling between neurons and oligodendrocytes has gained a lot of interest in recent years and it appears that oligodendrocytes also can release lactate. Recent studies displayed the involvement of oligodendrocytes in the pathogenesis of ALS (Lee et al., 2012; Kang et al., 2013; Philips et al., 2013). Healthy oligodendrocytes can metabolically support neurons by providing energy metabolites via monocarboxylate transporters (Fünfschilling et al., 2012; Lee et al., 2012; Morrison et al., 2013). In contrast, the transport of glycolytic substrates such as lactate from oligodendrocytes to neurons is found to be disrupted in SOD1^G93A^ mice spinal cord (Lee et al., 2012; Kang et al., 2013). Consistent with this, the expressions of the oligodendrocytic lactate transporter monocarboxylate transporter 1 (SLC16A1) was decreased in the spinal cords of patients with ALS and SOD1^G93A^ mice (Lee et al., 2012; Philips et al., 2013) suggesting abnormalities in lactate transfer.

In addition to astrocytes and oligodendrocytes, microglia have been shown to be involved in ALS pathogenesis (Beers et al., 2006; Boillée et al., 2006b). Microglia are immune cells within the CNS, similar to macrophages, which can either have a neuroprotective or a neurotoxic role during ALS progression. It is believed that microglia protect neurons early in the disease stage. However, as the disease progresses, activated microglia can be pro-inflammatory and neurotoxic (Appel et al., 2011; Dibaj et al., 2011). *In vivo* imaging studies demonstrated increased microglia activation and inflammatory activity in spinal cord during symptomatic stages in the SOD1^G93A^ mouse model (Dibaj et al., 2011). Reduced expression of mutant SOD1 in microglia delayed late disease progression and increased survival of SOD1^G37R^ mice (Boillée et al., 2006b). In conclusion, perturbations in glial cells can affect neurons and directly or indirectly can contribute to energy deficits in neurons. The studies discussed demonstrate the importance of non-neuronal cells in supporting neurons in various ways and indicate that abnormalities in glial cells can exacerbate the progression of the disease.

### High calorie diets in ALS

A number of studies have tried to correct the energy deficit using metabolic therapies in animal models and patients with ALS, with the hope that an improved metabolism can delay symptoms and extend survival in ALS (Dupuis et al., 2004; Zhao et al., 2006, 2012; Mattson et al., 2007; Miquel et al., 2012; Ari et al., 2014; Wills et al., 2014; Palamiuc et al., 2015; Tefera et al., 2016). A study by Dupuis et al. (2004) in SOD1^G86R^ mice found aberrations of energy metabolism such as reduction of adiposity, increased energy expenditure and lipolysis, and examined the effects of a high fat diet that consisted of regular chow supplemented with 21% (wt/wt) butter fat and 0.15% (wt/wt) cholesterol. A 20% increase in the mean survival of these mice compared to mice fed with control diet was demonstrated and motor neuron loss was reduced. Moreover, SOD1^G93A^ mice fed either a high fat diet (containing 47% fat, 38% carbohydrates and 15% protein) or with control diet (constituting 17% fats, 64% carbohydrates and 19% protein) starting at 6 weeks of age also showed an increase in survival and the onset of motor symptoms and weight loss were delayed significantly. While mice treated with control diet died by 180 days, high fat died treated mice survived until 220 days and some of them survived more than 270 days (Mattson et al., 2007). On a similar note, several studies have alluded that body mass index is strongly correlated with survival of patients with ALS (reviewed in Ngo et al., 2014). A prospective study of over a million people for about 14–28 years demonstrated that obese people have a 30–40% lower risk of developing ALS compared to those with a healthy weight (O'Reilly et al., 2013). Given the strong link found between low body mass index and decreased survival with ALS (Paganoni et al., 2011), a randomized double blind phase 2 clinical study was performed in small number of patients with ALS to test the safety and tolerability of high caloric diets, which were either high in carbohydrates or high in fat. Compared to an isocaloric control diet, the high carbohydrate-high calorie diet was safe, tolerable and effective in delaying weight loss and prolonging survival in the patients (Wills et al., 2014). Further large scale trials are needed to corroborate these promising effects. Also due to the small sample size, a beneficial effect of a high fat high calorie diet cannot yet be ruled out completely. On the other hand, high fat diets are linked with side effects such as brain inflammation, cognitive deficits, and anxiety and depressive behaviors (Zhang et al., 2005; Pistell et al., 2010; Dutheil et al., 2016). Together, these studies show the importance of adequate energy supply to slow the progression of ALS.

## Metabolic treatments in ALS

**Figure 1:**
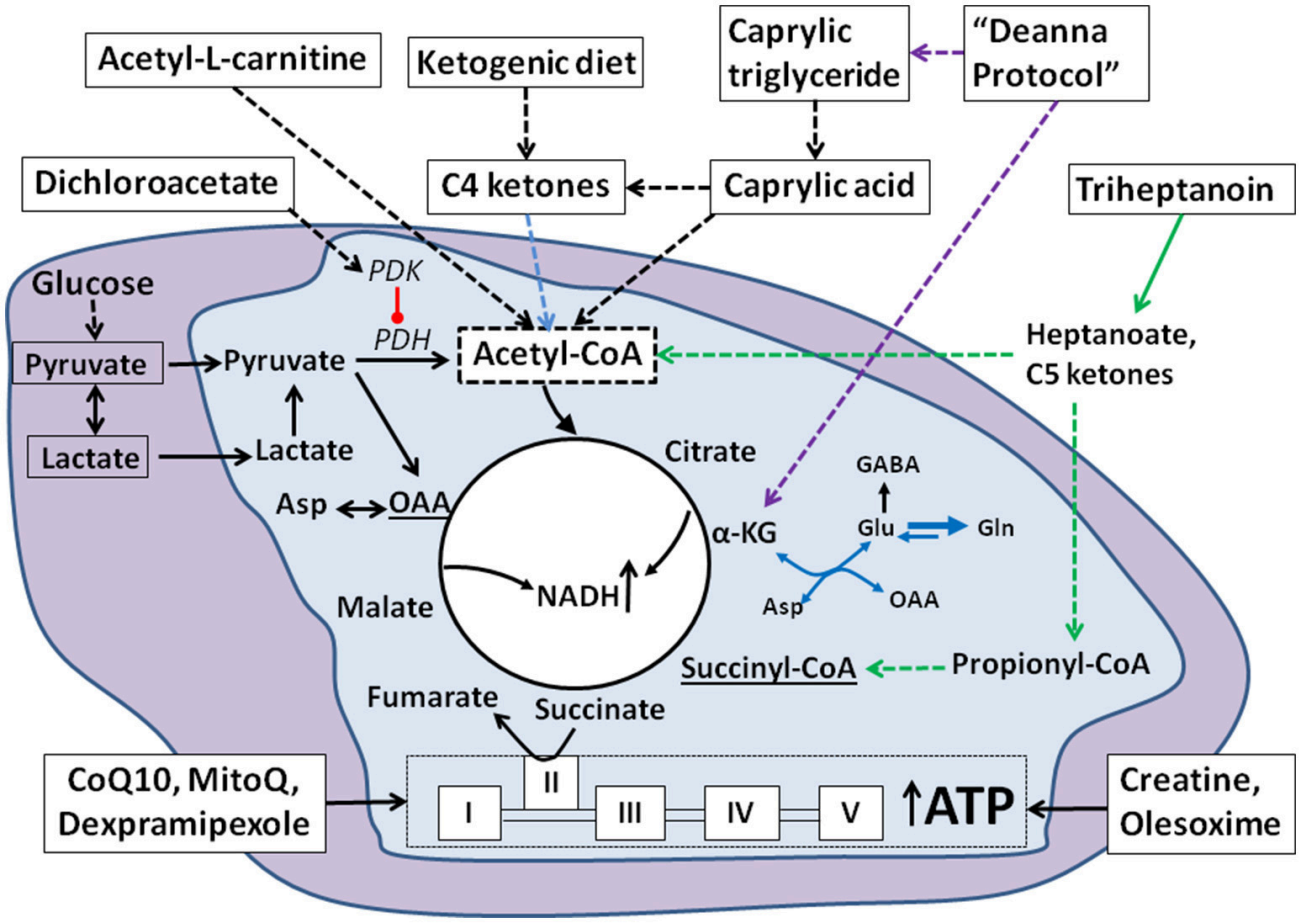
**Summary of cellular mechanisms of metabolic approaches in ALS**. Under normal physiological conditions, glucose is the main fuel for CNS or muscle cells and is metabolized via glycolysis into two molecules of pyruvate in the cytosol (purple background). Pyruvate can be converted by pyruvate dehydrogenase (PDH) into acetyl CoA in the mitochondria (light blue background), which enters the TCA cycle by condensing with oxaloacetate (OAA) to form citrate. Citrate gets further metabolized to generate NADH and ATP. Lactate is a glycolytic product that can be used to provide energy. According to the lactate shuttle hypothesis, lactate can also be transported into the mitochondria via monocarboxylate transporters and then get converted to pyruvate to enter the TCA cycle. In ALS, where energy demand is high and glucose metabolism is defective, alternative fuels can be provided by the ketogenic diet producing C4 ketones and the medium chain triglycerides (MCT). Among the MCTs, caprylic triglyceride is metabolized to C4 ketones and caprylic acid and triheptanoin provides heptanoate and C5 ketones, which all can be taken up from the blood and used by the CNS and muscle cells. The C4 and C5 ketones as well as caprylic acid are converted into acetyl-CoA and enter the TCA cycle. Increased C4 ketone levels can also activate astrocytes and facilitate conversion of glutamate into glutamine. This further provides more glutamine for the synthesis of GABA and less conversion of glutamate to aspartate (blue arrows, see section The Ketogenic Diet and Caprylic Triglyceride). Triheptanoin provides three molecules of heptanoate (green arrows), which can be converted into C5 ketones in the liver. Heptanoate and C5 ketones can enter the brain and muscle to be metabolized to acetyl-CoA and propionyl CoA. Propionyl CoA is an anaplerotic molecule that can be further converted into succinyl-CoA and enter into the TCA cycle. This restores (lost) C4 TCA cycle intermediate levels and promotes TCA cycling. Dichloroacetate is a PDH kinase inhibitor, which activates PDH thereby increasing the entry of pyruvate into the TCA cycle. Creatine provides high energy phosphates and stabilizes membranes and hence enhances mitochondrial function. The mitochondrial targeted antioxidants CoQ10, MitoQ and dexpramipexole promote mitochondrial energy generation. Olesoxime stabilizes mitochondrial membrane permeability transition pores. The Deanna protocol is a combination of supplements that can provide acetyl CoA and intermediates, such as α-ketoglutarate (α-KG) that feed into the TCA cycle (purple dashed arrows).

Given that defects in energy metabolism are evident in ALS, addressing these disturbances is a reasonable approach to improve quality of life and prolong survival in patients with ALS. Because of the impairments found in glucose metabolism together with the increased energy demand in neurons and muscles, additional fuel appears to be important. To increase energy uptake, additional alternative metabolic fuels for the CNS and muscle also appear warranted and could include ketones and medium chain fats, but also metabolic intermediates, such as pyruvate, lactate, α-ketoglutarate and others. Moreover, compounds that can enhance mitochondrial function to generate more energy are expected to ameliorate disease progression. This includes substances that improve mitochondrial function by inhibiting oxidative stress or stabilizing mitochondrial membranes. There are also supplements that provide energy substrates that can refill deficient C4 carbon TCA cycle intermediates (anaplerosis), which thereby will improve oxidative phosphorylation of any fuel in addition to providing alternative fuel. This includes triheptanoin and α-ketoglutarate. Consistent with these theoretical notions, alternative fuels, metabolic supplements and mitochondrial function enhancers have been investigated with the purpose of improving ALS symptoms and delaying disease progression in mouse models of ALS, cell cultures and in patients with ALS. These metabolic potential treatments include triheptanoin, ketogenic diet, caprylic triglyceride, dichloroacetate, the Deanna protocol, pyruvate, lactate, creatine, coenzyme Q10, MitoQ, dexpramipexole, acetyl-L-carnitine and olesoxime, which we will discuss below. Their mechanisms and effects are also summarized in Figure 1.

### Triheptanoin

Triheptanoin is an alternative fuel, which can also boost the metabolism of other fuels by refilling deficient C4 TCA cycle metabolites and is therefore ideal for disorders with high energy needs. Triheptanoin is a medium chain triglyceride (MCT) containing the odd chain C7 fatty acid heptanoate. After hydrolysis in the gastro-intestinal tract, medium chain fatty acids diffuse into cells, the blood stream and into mitochondria. This enhances their availability to various organs including the CNS and muscles. In contrast, long chain fatty acids are bound to plasma proteins and require transporter proteins for uptake into cells and mitochondria (Schönfeld and Wojtczak, 2016). When heptanoate is metabolized by β-oxidation, it generates two molecules of acetyl-CoA and one molecule of propionyl-CoA. In the liver, heptanoate can form the C5 ketones, β-ketopentanoate and β-hydroxypentanoate, which are released into the blood and can be taken up by neuronal and other cells through monocarboxylate transporters. Similar to heptanoate, the C5 ketones give rise to acetyl-CoA and propionyl-CoA. Propionyl-CoA enters the TCA cycle after it is carboxylated by propionyl-CoA carboxylase and then metabolized to succinyl-CoA, thereby (re-) filling the (deficient) C4 TCA cycle intermediate pool (reviewed in Brunengraber and Roe, 2006). C4 TCA cycle intermediates contribute to the formation of oxaloacetate, which is needed to condense with acetyl-CoA to citrate. Thus, overall heptanoate and C5 ketones provide additional alternative fuel to form acetyl-CoA and by increasing the levels of oxaloacetate improve acetyl-CoA entry into the TCA cycle, required for aerobic ATP production. The ability of triheptanoin to provide both acetyl-CoA and propionyl-CoA makes it an ideal metabolic treatment for disorders with deficient levels of TCA cycle intermediates and impairments in energy production. For instance, triheptanoin alleviated metabolic disturbances associated with several neurological and neuromuscular disorders and improved symptoms in models of epilepsy (Willis et al., 2010; Thomas et al., 2012; Hadera et al., 2014), Canavan disease (Francis et al., 2014), autism disorders (Park et al., 2014) and Alzheimer's Disease (Aso et al., 2013) as well as patients and a mouse model of glucose transporter 1 deficiency (Marin-Valencia et al., 2013; Pascual et al., 2014; Mochel et al., 2016), and patients with Huntington's disease (Mochel et al., 2010; Adanyeguh et al., 2015). In chronically “epileptic” mice it was shown that triheptanoin partially restores reduced TCA cycle intermediate and metabolite levels (Willis et al., 2010; Hadera et al., 2014). Cognisant of the positive effects of triheptanoin in other brain and neuromuscular disorders and its mechanism of action, we hypothesized that treatment with triheptanoin, being an alternative substrate to glucose provides degenerating cells with much needed energy, which can delay cell death and thereby the onset of motor symptoms in the ALS mouse model. Thus we recently investigated the effects of triheptanoin in the hSOD1^G93A^ mice (Tefera et al., 2016).

We first showed that in the gastrocnemius muscle of the hSOD1^G93A^ mice, gene expression of enzymes involved in the glycolysis, TCA cycle and anaplerotic pathways such as glyceraldehyde-3-phosphate dehydrogenase, pyruvate, 2-oxoglutarate and succinate dehydrogenases, at earlier (10 weeks) and at later symptomatic stages (25 weeks) of the disease was reduced. Downregulation of these metabolic enzymes in muscle even at early stages where grip strength was still intact suggests that metabolic defects in the energy producing pathways could contribute to disease progression in ALS (Tefera et al., 2016). We also found reduced activities of 2-oxoglutarate dehydrogenase enzyme at the late stage of the disease (25 weeks) indicating abnormalities in the TCA cycle pathway. We then showed that treatment of hSOD1^G93A^ mice with triheptanoin (where 35% of calories were from this oil, 17% w/diet w) from P35 until P70 prevented downregulation of the enzymes such as succinate dehydrogenase, glutamic pyruvic transaminase 2 and the propionyl carboxylase β subunit compared to mice treated with control diet. Similarly, treatment with triheptanoin protected against motor neuron loss by 33% in the lumbar spinal cord (Tefera et al., 2016). It also delayed the onset of motor symptoms which was shown by improvements in grip strength and rotarod tests. It is believed that the neuroprotective effects of triheptanoin due to increased ATP generation contribute to improvements in motor function. In addition, increased ATP generation in remaining neurons and muscle is likely to also benefit motor function. Please note that our study was underpowered to determine changes in survival. It remains to be seen if triheptanoin is effective in other rodent ALS models and if it can be tolerated by patients with ALS.

### The ketogenic diet and caprylic triglyceride

Ketogenic diets consist of extremely high amounts of fats, while carbohydrates and proteins are restricted. They are successfully used for the treatment of childhood epilepsies, where several controlled clinical trials have now shown efficacy (Martin et al., 2016) and there is also some efficacy against seizures in adults. Ketogenic diets are now being tested in a variety of disorders, including some characterized by neurodegeneration, such as Alzheimer's Disease (Van der Auwera et al., 2005; Henderson et al., 2009) and traumatic brain (Prins et al., 2005; Appelberg et al., 2009; Hu et al., 2009b; Deng-Bryant et al., 2011) and spinal injury (Streijger et al., 2013). Administration of ketones after controlled cortical impact injury in young rats reduced lesion volume, attenuated motor and cognitive deficits, reduced cellular apoptotic markers, and improved cerebral ATP levels (Prins et al., 2005; Appelberg et al., 2009; Hu et al., 2009a,b; Deng-Bryant et al., 2011). Also, BHB was able to rescue neurons from degeneration, alleviate motor deficits against 1-Methyl-4-phenyl-1,2,3,6-tetrahydropyridine (MPTP) toxicity, and improve mitochondrial ATP generation (Tieu et al., 2003). Similarly, it improved cerebral energy metabolism in ischemic rat brain (Suzuki et al., 2001) and prevented glutamate mediated neuronal toxicity and lipid peroxidation (Mejía-Toiber et al., 2006). While on a ketogenic diet, blood glucose levels remain consistently in the lower part of the normal range and the liver produces the C4 ketones, β-hydroxy butyrate (BHB) and aceto-acetate, which are secreted into the blood. If C4 ketones are available, the CNS will use C4 ketones as fuel preferably to glucose (Courchesne-Loyer et al., 2016).

In SOD1^G93A^ mice, a ketogenic diet was shown to protect against the loss of motor neurons in the spinal cord and to delay the onset of behavioral symptoms (Zhao et al., 2006). BHB was also able to increase the rate of ATP generation in mitochondria isolated from the spinal cord of SOD1^G93A^ mice as well as in cultured spinal cord neurons from these mice in the presence of rotenone (Zhao et al., 2006). Mechanisms by which the ketogenic diet modifies disease progression are probably multifold (Masino and Rho, 2012). Most likely the use of C4 ketones as alternative fuels as well as their anti-oxidant and anti-inflammatory effects plays important roles. Moreover, C4 ketones can alter metabolism in the brain. It has been hypothesized that ketones activate astrocytes and hence increases the conversion of glutamate to glutamine by glutamine synthetase thereby reducing the availability of glutamate in the synapse (Yudkoff et al., 2008). This reduces glutamate excitotoxicity, which is one of the mechanisms most likely involved in the pathogenesis of ALS (Rothstein et al., 1990). Moreover, according to the glutamate-glutamine-GABA cycle, astrocytes release glutamine to neurons and inhibitory neurons convert it to GABA. Thus, more glutamine in astrocytes may also lead to increased availability of glutamate for the synthesis of GABA, the primary inhibitory neurotransmitter in the brain (Yudkoff et al., 2008). It is also hypothesized that this leads to less glutamate available to produce aspartate, another excitatory neurotransmitter in the brain (Yudkoff et al., 2008). Cortical hyperexcitability due to reduced intracortical inhibition has been shown in patients with ALS and preceded the onset of ALS symptoms (Vucic and Kiernan, 2006; Vucic et al., 2008). Reductions in GABA levels found in patients with ALS in motor cortex compared to healthy controls (Foerster et al., 2012, 2013) may contribute to cortical hyperexcitability. Similarly, in the cortex of wobbler mouse model of ALS, reduced GABA mediated inhibition in the layer 5 pyramidal neurons as a result of loss of GABAergic interneurons was shown, resulting in cortical excitability (Nieto-Gonzalez et al., 2011).

The MCT of eight carbon octanoate, caprylic triglyceride, has also been investigated (Zhao et al., 2012). After hydrolysis of the ester bonds, octanoic (caprylic) acid can be metabolized quickly by β-oxidation to acetyl CoA and C4 ketone bodies to supply the TCA cycle. As mentioned above, one advantage of medium chain fatty acids is that they are able to easily diffuse through plasma as well as mitochondrial membranes and do not require transport proteins (Schönfeld and Wojtczak, 2016). Thus, MCTs can serve as a fast fuel especially in conditions with decreased uptake of glucose. In SOD1^G93A^ mice, feeding 10% (w/w) caprylic triglyceride improved motor performance in the rotarod test, protected against motor neuron loss and increased oxygen consumption in mitochondria isolated from the spinal cord (Zhao et al., 2012). Please note that this MCT dose is not very high and blood ketone levels were unchanged in the presymptomatic phase and only about doubled in the postsymptomatic phase in SOD1^G93A^ mice. In comparison while on ketogenic diet the levels quadrupled in the same mouse strain (Zhao et al., 2006). This may indicate that octanoate itself may be sufficient for neuroprotection and ketone formation may not be necessary.

Disappointingly, neither the ketogenic diet nor caprylic triglyceride improved the survival of SOD1^G93A^ mice (Zhao et al., 2006, 2012). Also, it is important to note that in other studies octanoate limited glucose metabolism in rodents (Wlaz et al., 2012; McDonald et al., 2014). Given that glucose utilization is compromised in ALS, further inhibiting glucose metabolism may worsen disease progression. Last, ketogenic diets are associated with loss of body weight, which in ALS can worsen disease progression. Thus, these apparently successful metabolic approaches in this ALS mouse model cannot be fully recommended for ALS patients.

### The deanna protocol

The Deanna protocol is a combination of nutritional supplements containing energy supplements, vitamins, and antioxidants such as glutathione, nicotinamide adenine dinucleotide (NADH), coenzyme Q10 (CoQ10) and ubiquinol in addition to MCTs if tolerated. It was claimed to have beneficial effects in anecdotal reports (Fournier et al., 2013). The effects of the main constituents of the supplement, including arginine α-ketoglutarate (AAKG), MCT rich in caprylic triglyceride and GABA, were tested in SOD1^G93A^ mice (Ari et al., 2014). AAKG provides α-ketoglutarate, which is taken up by sodium-dependent transport carriers into synaptosomes and can be incorporated into the TCA cycle (Shank and Campbell, 1981, 1984). In addition, the medium chain fats can be rapidly metabolized and easily diffuse into mitochondria to provide acetyl CoA. Ari and colleagues showed that this simplified metabolic treatment delayed motor symptoms and increased survival in SOD1^G93A^ mice (Ari et al., 2014). In our opinion, the true effects of the Deanna protocol in patients with ALS are worthwhile to be studied. However, this will be very difficult, because most patients take dietary supplements and vitamins.

### Dichloroacetate

Dichloroacetate (DCA) is a pyruvate dehydrogenase (PDH) kinase inhibitor (Whitehouse et al., 1974). Inhibition of PDH kinase leads to less phosphorylation of PDH resulting in activation of this enzyme that catalyzes the conversion of pyruvate to acetyl CoA (McAllister et al., 1973). Thus, DCA facilitates oxidation of glucose and generation of ATP in the mitochondria (Itoh et al., 2003). The effects of DCA were examined in the SOD1^G93A^ mouse model of ALS (Miquel et al., 2012). DCA prevented the toxicity of astrocytes to motor neurons in neuron-astrocyte co-cultures from SOD1^G93A^ rat spinal cord. In addition, when DCA was administered to SOD1^G93A^ mice starting at 10 weeks; it reduced motor neuron loss in the lumbar spinal cord by 25%, delayed onset of ALS motor symptoms in a grip strength test and improved survival (Miquel et al., 2012). The mechanism by which it modifies disease progression is believed to be improved astrocytic TCA cycling and mitochondrial function (Miquel et al., 2012). In another similar study by Palamiuc et al. (2015) in SOD1^G86R^ mice, the protective effects of DCA were corroborated. In this study, DCA improved grip strength performance, prevented downregulation of mitochondrial genes, and protected against muscle denervation. At doses used in metabolic studies, DCA was well tolerated in patients with brain cancers (Dunbar et al., 2014). Its safety, tolerability and efficacy in ALS patients remain to be studied.

### Pyruvate

Pyruvate is a glycolytic metabolite that can enter the TCA cycle following its conversion into acetyl CoA. It was shown to be neuroprotective in cultured mouse striatal neurons against N-methyl D-aspartate (NMDA) and H_2_O_2_ mediated toxicity (Desagher et al., 1997; Maus et al., 1999) and in cortical neuronal cultures subjected to oxygen-glucose deprivation by improving cellular energy metabolism, reducing reactive oxygen species levels and anti-inflammatory mechanisms (Shen et al., 2010; Pan et al., 2012). Pyruvate's ability to protect cortical neurons against H_2_O_2_ toxicity involves monocarboxylate transporters (Nakamichi et al., 2005). In juvenile rat hippocampal slices, pyruvate administration was able to restore ATP levels and rescue neurons against NMDA mediated excitotoxicity (Izumi and Zorumski, 2010). Pyruvate treatment reduced infarct size and improved neurological functions in a rat middle cerebral artery occlusion model of ischemia (Yu et al., 2005). It also improved cerebral oxidative metabolism and neurological outcomes in rats following cortical contusion injury (Fukushima et al., 2009; Moro and Sutton, 2010), protected against MPTP induced degeneration of neurons and reduced oxidative stress in a mouse model of Parkinson's disease (Huh et al., 2011; Satpute et al., 2013), and reduced cognitive deficits in a mouse model of Alzheimer's disease (Isopi et al., 2015).

In the SOD1^G93A^ mouse model of ALS, pyruvate (1000 mg/kg/week i.p.) slowed disease progression and also improved motor symptoms in a rotarod test and survival by 10.5% when it was administered starting at the onset of disease (Park et al., 2007). However, in a similar study performed to evaluate the effects of pyruvate with increased frequency of dosing (500 mg/kg i.p., 6 days a week) in SOD1^G93A^ mice, pyruvate was not able to alter the onset of the disease or survival of mice (Esposito et al., 2007). Esposito and colleagues hypothesized that their higher levels of pyruvate may not lead to larger benefit due to pyruvate's U-shaped dose-response curve (Esposito et al., 2007). The different outcomes may therefore be explained by the use of different dosing and different mouse strains.

### Lactate

Lactate is the end product of the glycolytic pathway (Rogatzki et al., 2015) and also can be utilized as an energy fuel following its conversion into pyruvate and subsequent oxidation in the mitochondria (Schurr, 2008). Studies have demonstrated the role of lactate as a neuroprotective energy substrate in brain disorders such as cerebral ischemia (Schurr et al., 2001; Berthet et al., 2009; Horn and Klein, 2013; Castillo et al., 2015) and traumatic brain injury (TBI) (reviewed in Brooks and Martin, 2014; Carpenter et al., 2015; Patet et al., 2016). Early administration of lactate in rat organotypic hippocampal slices after oxygen-glucose deprivation protected against neuronal death, decreased lesion size and showed better neurologic outcomes in a mouse model of middle cerebral artery occlusion (Berthet et al., 2009, 2012). Lactate has also been shown to provide neuroprotection against glutamate mediated toxicity in rat hippocampal slices (Schurr and Gozal, 2011), in mouse cortical neuron cultures (Jourdain et al., 2016) and in rat cortex *in vivo* (Ros et al., 2001). In addition, exogenous administration of lactate reduced brain lesion size in a rat controlled cortical impact models of TBI (Alessandri et al., 2012) and in patients with severe TBI (Bouzat et al., 2014). Similarly, lactate infusion prevented intracranial hypertensive episodes in patients with severe TBI (Ichai et al., 2009, 2013; Bouzat et al., 2014), and attenuated cognitive deficits in rat lateral fluid percussion injury (Rice et al., 2002; Holloway et al., 2007) and in patients with mild TBI (Bisri et al., 2016).

The mechanism of lactate was linked to its ability to improve cerebral energy metabolism and preserve ATP levels in injured brain (Holloway et al., 2007; Gallagher et al., 2009; Bouzat et al., 2014; Jourdain et al., 2016). In addition to its effect as an energy fuel, lactate can function as a signaling molecule by activating hydrocarboxylic acid receptor 1 (HCA1) receptors (Castillo et al., 2015). ATP produced from lactate was also shown to act on the P2Y receptors (Jourdain et al., 2016). Lactate can be used either directly following its cerebral uptake or indirectly as a gluconeogenic precursor (Glenn et al., 2015a,b). Using lactate as an energy fuel for neurons with energy deficit in diseases such as ALS may improve symptoms, especially if reductions of astrocytic lactate transporters found in SOD1^G93A^ mice (Ferraiuolo et al., 2011) are not affecting neuronal lactate uptake.

### Creatine

Creatine is phosphorylated by ATP to phosphocreatine, which is another high energy phosphate in addition to ATP. Sufficient amounts of creatine are therefore important to prevent energy depletion. It also stabilizes mitochondrial membranes by binding to phospholipids (Persky and Brazeau, 2001). It has demonstrated beneficial effects in treating energy impairments in several neurodegenerative disorders such as Alzheimer's disease, Parkinson's disease and Huntington's disease (reviewed by Adhihetty and Beal, 2008). Early studies in ALS showed that administration of creatine to SOD1^G93A^ mice prevented loss of motor neurons, protected neurons from oxidative damage in the spinal cord, and improved motor performance and survival of mice in a dose dependent manner (Klivenyi et al., 1999). Also, long term supplementation of creatine in SOD1^G93A^ mice was able to reduce NMDA induced release of glutamate in the cerebral cortex (Andreassen et al., 2001b). Similarly, treatment with creatine prevented depletion of ATP in spinal cord and cerebral cortex of SOD1^G93A^ mice (Browne et al., 2006). Although preclinical studies were promising, several clinical trials failed to show significant beneficial effects in improving the progression of the disease or survival of patients (Groeneveld et al., 2003; Shefner et al., 2004; Rosenfeld et al., 2008).

### Coenzyme Q

Coenzyme Q is an electron acceptor in the electron transport chain in mitochondria. It is a powerful antioxidant that scavenges free radicals. Because of its effect in mitochondrial disorders, coenzyme Q has been tried in the SOD1^G93A^ mouse model of ALS (Matthews et al., 1998) and in patients with ALS (Kaufmann et al., 2009). The idea is to promote activation of enzymes involved in the electron transport chain which might be defected due to reduced coenzyme Q in ALS. Coenzyme Q was neuroprotective against 3-nitropropionic induced striatal lesions and it increased the life span of SOD1^G93A^ mice (Matthews et al., 1998). However, the promising effects of Coenzyme Q in animal experiments were not replicated in phase II clinical trials (Kaufmann et al., 2009).

### MitoQ

MitoQ is an antioxidant which accumulates in mitochondria (Kelso et al., 2001). It is a derivative of coenzyme Q that promotes the uptake of endogenous coenzyme Q and improves mitochondrial function (Tauskela, 2007). Cassina et al. (2008) showed that mitochondrial function is impaired in the spinal cords of symptomatic SOD1^G93A^ rats, specifically mitochondrial oxygen consumption by the electron transport chain. In addition, SOD1^G93A^ rat astrocytes showed signs of oxidative stress. Therefore, they used MitoQ to target antioxidants to the mitochondria. They demonstrated that pretreatment of cultured SOD1^G93A^ astrocytes with MitoQ was able to reduce the formation of superoxide and peroxynitrite radicals, protect motor neurons against oxidative damage and improve the ability of mitochondria to generate ATP (Cassina et al., 2008). Consistent with this, in a similar study in the SOD1^G93A^ mouse model, treatment with MitoQ at the onset of disease also delayed the decline of mitochondrial function in the spinal cord, protected against motor neuron loss and astrogliosis in the spinal cord. Moreover, it preserved the integrity of the neuromuscular junction, improved hind limb grip strength as well as the life span of mice (Miquel et al., 2014), raising the hope that MitoQ may be effective in ALS patients. On the other hand, in a study with 128 Parkinson's Disease patients, 40 and 80 mg MitoQ per day was associated with dose-dependent increased incidence of nausea and vomiting (Snow et al., 2010). Also, there was no change in progression of disease on a Parkinson Disease Rating Scale.

### Dexpramipexole

Dexpramipexole, an enantiomer of pramipexole, is believed to increase ATP generation by improving efficiency of oxidative phosphorylation as well as reducing oxidative damage of mitochondria (Gribkoff and Bozik, 2008). *In vitro* studies showed that it is able to promote metabolic efficiency of injured cells and scavenging of free radicals in the mitochondria (Danzeisen et al., 2006; Alavian et al., 2012). In another study, dexpramipexole prevented H_2_O_2_ mediated cell death and prevented the generation of mitochondrial reactive oxygen species in neuroblastoma cells (Ferrari-Toninelli et al., 2010). In the SOD1^G93A^ mouse model, dexpramipexole preserved motor function and prolonged life span (Danzeisen et al., 2006). Due to these beneficial effects *in vitro* and *in vivo*, dexpramipexole was investigated in a double blind randomized phase 2 clinical trial in patients with ALS, showing that treatment for 12 months was safe and well tolerated (Cudkowicz et al., 2011). Dexpramipexole also prevented functional decline in a dose dependent manner compared to placebo (Cudkowicz et al., 2011). However, despite the promising effects in this study, a phase 3 clinical trial failed to show improvements in disease symptoms (Cudkowicz et al., 2013). Dexpramipexole's failure was linked to lack of prior rigorous preclinical testing. Studies performed after the last clinical trial, in induced pluripotent stem cells (iPSCs) derived from patients with ALS (Yang et al., 2013), in rat primary cortical neurons transfected with human mutant TDP-43 as well as in the SOD1^G93A^ mouse model (Vieira et al., 2014) showed no beneficial effect.

### Acetyl-L-carnitine

Acetyl-L-carnitine supplies L-carnitine, which is important in the transport of long chain fatty acids across mitochondrial membranes (Fritz and Yue, 1964). In addition, the acetyl moiety can be transferred to provide acetyl CoA. Therefore, acetyl-L-carnitine improves cerebral oxidative energy production (Zanelli et al., 2005) and fatty acid oxidation in muscles (Siliprandi et al., 1965). It has shown beneficial effects in models of traumatic brain (Scafidi et al., 2010) and spinal cord injury (Zhang et al., 2015), cerebral ischemia (Shuaib et al., 1995; Jalal et al., 2010), stroke (Lolic et al., 1997) and Alzheimer's disease (Virmani et al., 2001) and patients with Alzheimer's disease (Spagnoli et al., 1991; Pettegrew et al., 1995). In primary motor neuron cultures from rats, acetyl-L-carnitine was found to protect against kainate and NMDA-induced toxicity (Bigini et al., 2002). In light of this, a randomized double blind placebo-controlled trial was performed in small number of patients with ALS to investigate the effects of add-on acetyl-L-carnitine to riluzole vs. riluzole only treatment. Patients tolerated the combination well and had a better score in the ALS Functional Rating Scale (Beghi et al., 2013). More *in vivo* studies in ALS models and a larger phase III trial are now needed to prove efficacy.

### Olesoxime

Olesoxime is an experimental compound believed to stabilize mitochondrial permeability transition pores (Bordet et al., 2007). *In vitro* studies showed increased motor neuron survival in a motor neuron degeneration model in rats (Bordet et al., 2007). In addition, treatment of SOD1^G93A^ transgenic mice with olesoxime improved motor function in the hanging grid test, delayed the onset of body weight loss and prolonged survival by 10% (Bordet et al., 2007). Olesoxime was also shown to delay denervation of muscle and decrease activation of microglia and motor neuron death in the lumbar spinal cord (Sunyach et al., 2012). However, a phase II-III double blind, randomized, placebo-controlled clinical trial failed to show efficacy in patients with ALS (Lenglet et al., 2014). Similar to dexpramipexole, absence of rigorous evaluation in preclinical studies may have led to its failure. In iPSCs derived from patients with ALS, olesoxime showed inconsistent effects (Yang et al., 2013).

### Others

Other compounds that directly or indirectly affect energy metabolism such as sirtuins prevent fragmentation of mitochondria and promote an increase in ketone levels and are potential therapeutic targets in ALS (Körner et al., 2013a; Pasinetti et al., 2013). Resveratrol, an antioxidant, which is believed to increase sirtuin 1 expression, was able to delay onset of symptoms, prevent motor neuron loss and extend survival when treatment was initiated at early stages of the disease in hSOD1^G93A^ mice (Mancuso et al., 2014; Song et al., 2014). Moreover, dysregulation of AMP-activated protein kinase (AMPK), an energy sensor which is induced by energy depletion was also linked to ALS (reviewed in Perera and Turner, 2016). AMPK was shown to be activated in TDP-43 mice spinal cord (Perera et al., 2014) and reducing its activity prevented loss of motor neurons in *Caenorhabditis elegans* models of ALS (Lim et al., 2012). In general, substances that can counteract energy depletion through modulation of the energy producing pathways could help in delaying disease progression.

## Conclusions

Of the metabolic approaches discussed above, creatine, dexpramipexole, olesoxime, MitoQ and Coenzyme Q have been investigated in randomized clinical trials (summarized in Table 1). All failed to deliver beneficial effects except acetyl-L-carnitine which showed improved ALS Functional Rating Scale in small number of patients in a phase II clinical trial. The effects of ketogenic diet, caprylic triglyceride, DCA, the Deanna protocol and triheptanoin in patients with ALS remains to be seen. It is critical to note that although numerous (around 50) compounds have been investigated in randomized clinical trials, almost all of them failed to show efficacy except riluzole, the only approved drug so far (Mitsumoto et al., 2014). On the other hand, analysis of preclinical therapeutic trials in hSOD1^G93A^ mouse model of ALS revealed that publication bias and poorly designed experiments such as absence of randomization and blinding, lack of adequate statistical power, and variations within the animal models may lead to over reporting of false positive results (Benatar, 2007; Scott et al., 2008; Perrin, 2014). In addition, methodological flaws in clinical trials could also lead to failure to find efficacy of treatments (Mitsumoto et al., 2014). Therefore, it is of utmost importance to investigate future potential treatments in adequately designed preclinical and clinical trials.

**Table 1:** **Summary of metabolic approaches in ALS and their effects in models of ALS and patients with ALS**.

**Metabolic therapy**	**Proposed mechanisms**	**Effects on ALS models**	**Effects on clinical trials**
Acetyl-L-carnitine	Provides acetyl-CoA, improves fatty acid transport	Protected against kainate and NMDA toxicity *in vitro* (Bigini et al., 2002)	Improved ALSFR scale in phase II trial (Beghi et al., 2013)
Caprylic triglyceride	Provides acetyl-CoA and ketones	Delayed motor symptoms, prevented motor neuron loss, no effect on survival (Zhao et al., 2012)	ND
Creatine	Provides high energy phosphates and stabilizes mitochondrial membranes	Prevented motor neuron loss, improved motor performance and survival (Klivenyi et al., 1999) Prevented ATP depletion in brain and spinal cord (Browne et al., 2006) Reduced glutamate increase (Andreassen et al., 2001a)	No efficacy in phase II-III clinical trials (Groeneveld et al., 2003; Shefner et al., 2004; Rosenfeld et al., 2008)
CoQ10	Antioxidant, improves mitochondrial function	Neuroprotective in animal models, increased survival (Matthews et al., 1998)	No efficacy in Phase II clinical trial (Kaufmann et al., 2009)
Deanna protocol	Provides acetyl-CoA, ketones and α-KG	Delayed motor symptoms, increased survival (Ari et al., 2014)	ND
Dexpramipexole	Aid ATP generation Scavenge free radicals	Improved motor function, prolonged survival (Danzeisen et al., 2006)	Improved ALSFR scale in Phase II clinical trial (Cudkowicz et al., 2011) No benefit in Phase III clinical trial (Cudkowicz et al., 2013)
Dichloroacetate	Inhibits PDH kinase	Delayed motor symptoms, prevented motor neuron loss, increased survival (Miquel et al., 2012; Palamiuc et al., 2015)	ND
Ketogenic diet	Provides C4 ketones	Delayed motor symptoms, no effect on survival (Zhao et al., 2006)	ND
MitoQ	Antioxidant, improves mitochondrial function	Prevented motor neuron loss, improved motor function and survival (Cassina et al., 2008; Miquel et al., 2014)	ND
Olesoxime	Stabilizes mitochondrial permeability transition pore	Delayed motor symptoms, increased survival (Bordet et al., 2007)	No efficacy in phases II-III clinical trials (Lenglet et al., 2014)
Pyruvate	Provides acetyl-CoA, antioxidant	Slowed motor symptoms, increased survival (Park et al., 2007) No effects on disease progression (Esposito et al., 2007)	ND
Triheptanoin	Anaplerotic, provides acetyl-CoA, propionyl CoA and C5 ketones	Delayed motor symptoms, prevented motor neuron loss (Tefera et al., 2016)	ND

*ND, not determined*.

In conclusion, many studies demonstrated that metabolic disturbances are evident in patients with ALS and animal models. Only, a few studies have investigated the effects of metabolic treatments in ALS disease progression and survival. The therapeutic effects of these compounds in animal models and *in vitro* studies either in delaying motor symptoms, protecting against motor neuron loss and/or improving survival suggest that further studies should be performed to characterize abnormalities in energy metabolism in ALS and explore potential metabolic therapies.

## Author contributions

TT and KB designed the review. TT drafted the review. KB revised critically for important intellectual content. TT and KB approved this to be published.

## Funding

We are grateful for funding from NHMRC (KB, 014068) and UQ International (TT).

### Conflict of interest statement

KB applied for a patent for use of triheptanoin in ALS. The other author declares that the research was conducted in the absence of any commercial or financial relationships that could be construed as a potential conflict of interest.
